# Ultraviolet Irradiation Enhances the Microbicidal Activity of Silver Nanoparticles by Hydroxyl Radicals

**DOI:** 10.3390/ijms21093204

**Published:** 2020-04-30

**Authors:** Shingo Nakamura, Naoko Ando, Masahiro Sato, Masayuki Ishihara

**Affiliations:** 1 Division of Biomedical Engineering, National Defense Medical College Research Institute, Saitama 359-8513, Japan; naoandokoro@gmail.com (N.A.); ishihara@ndmc.ac.jp (M.I.); 2 Section of Gene Expression Regulation, Frontier Science Research Center, Kagoshima University, Kagoshima 890-8544, Japan; masasato@m.kufm.kagoshima-u.ac.jp

**Keywords:** healthcare workers, hydroxyl radical, medical application, microbicidal activity, silver nanoparticles (AgNPs), ultraviolet (UV) irradiation

## Abstract

It is known that silver has microbicidal qualities; even at a low concentration, silver is active against many kinds of bacteria. Silver nanoparticles (AgNPs) have been extensively studied for a wide range of applications. Alternately, the toxicity of silver to human cells is considerably lower than that to bacteria. Recent studies have shown that AgNPs also have antiviral activity. We found that large amounts of hydroxyl radicals—highly reactive molecular species—are generated when AgNPs are irradiated with ultraviolet (UV) radiation with a wavelength of 365 nm, classified as ultraviolet A (UVA). In this study, we used electron spin resonance direct detection to confirm that UV irradiation of AgNPs produced rapid generation of hydroxyl radicals. As hydroxyl radicals are known to degrade bacteria, viruses, and some chemicals, the enhancement of the microbicidal activity of AgNPs by UV radiation could be valuable for the protection of healthcare workers and the prevention of the spread of infectious diseases.

## 1. Introduction

The microbicidal effects of silver have widely been known and are even apparent at low concentrations. Silver has antibacterial action against a wide range of species, and its toxicity to human cells is considerably lower than that toward bacteria [1,2,3,4,5]. Berger et al. [1] reported that approximately 1 µg/mL of silver ions is effective for suppressing the growth of many types of bacteria, including *Escherichia coli* (*E. coli*), Staphylococci, Providencia, Serratia, and *Pseudomonas aeruginosa*. Ip et al. [2] demonstrated that several silver ion-containing vulneraries covering materials exhibited antibacterial activity against methicillin-resistant *Staphylococcus aureus*. Although the details of the mechanism by which silver produces its antibacterial activity remains largely unknown, the production of silver ions is thought to be one of the main factors [6]. Silver ions react with enzymes that require thiol (–SH) groups for the cysteine residue of their active sites; such enzymes are succinate dehydrogenase and nicotinamide adenine dinucleotide (NADH)-cytochrome b, which inhibit the metabolic pathways needed for the survival of bacteria [7].

“Nano” metallic particles are defined as those with sized 1 to 100 nm [8]. Metallic nanoparticles exhibit a size-specific property known as surface plasmon resonance (SPR) [9]. Due to their lager surface area, silver nanoparticles (AgNPs) enable the continuous release of silver ions at low concentrations, producing antibacterial action against a wide range of species [10]. Recently, AgNPs have also been shown to have antiviral activity. The size of the AgNPs is essential to their antiviral effects, as AgNPs act on the surface of the virus and physically inhibit contact with host cells [11,12,13]. Lara et al. [14] demonstrated that AgNPs of less than 10 nm preferentially adsorb the envelope of human immunodeficiency virus-1 (HIV-1) and prevent the viral infection of host cells. Gaikwad et al. [15] reported that AgNPs of 7–20 nm exhibited antiviral effects against herpes simplex virus types 1 and 2, and human parainfluenza virus type 3.

We previously reported on the possibility of using AgNPs to prevent infection of healthcare workers [16]. In this report, we describe a novel, convenient method for the synthesis of AgNPs of controlled size and demonstrate that chitin or chitosan-based materials act as excellent stabilizers of the AgNPs [17,18,19]. These composite materials, when coated with AgNPs, can exhibit strong microbicidal activity [20] and virus inactivation activity against the influenza A virus subtype H1N1 [21]. In addition to generating free silver ions, it has been suggested that AgNPs may yield reactive oxygen species (ROS), leading to oxidative stress [22]. Thus, the AgNPs could be widely used as antibacterial and antiviral materials in a variety of fields using clothing (doctor and nurse uniforms, security protection coats, masks, and counter cloths), plastics (gloves), and papers. We suggest that AgNPs may be valuable materials, owing to their bactericidal activity, when the levels of ROS produced from AgNPs are well-controlled.

Metal nanoparticles, including AgNPs, exhibit a property known as SPR. The absorption and scattering of light by AgNPs is highly efficient. The excitation of metal atoms on a metal surface stimulated by photons with a specific wavelength causes a collective oscillation of conduction electrons. SPR-based absorption spectroscopy depends on the size, shape, and composition of the metal surface, and on changes in response to alterations of the metal surface [23]. This approach can be applied to the detection of structural changes caused by the adsorption of substances onto metals, and several biosensing devices employ this principle. In this study, we investigated the generation of radicals following ultraviolet (UV) irradiation of AgNPs and the effect of the generated radicals as bactericides. Taking this phenomenon as a hint, we hypothesized that radicals are generated when AgNPs are irradiated with specific wavelengths of UV radiation that cause rapid oscillations in electron density and collisions in subsequent low-energy ion atoms.

## 2. Results and Discussion

### 2.1. UV Irradiation-Induced Generation of Radicals

**Figure 1:**
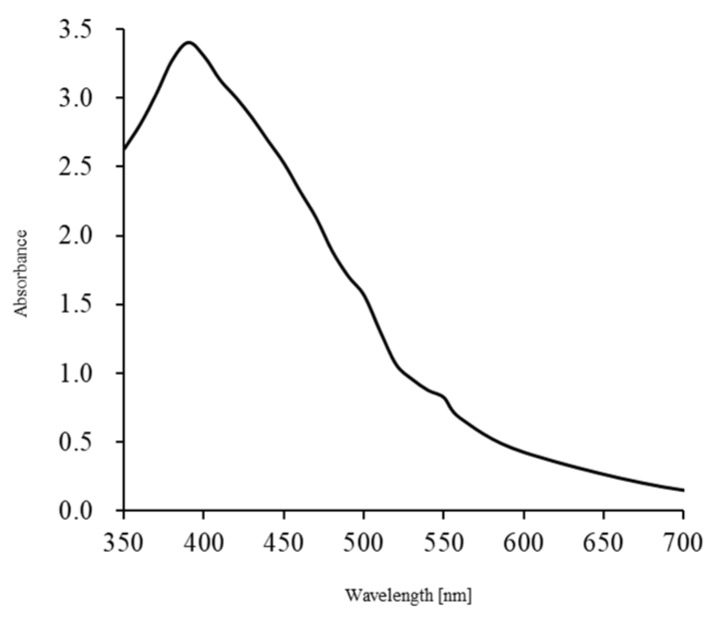
UV–Vis spectra of silver nanoparticles (AgNPs) used in this study. UV–Vis spectra from AgNPs in suspension peaked at 390 nm, which is considered as a representative pattern of the spherical AgNPs [17,18,19].

The silver nanoparticles used in this study were prepared using a method we previously reported [20]. The resulting AgNPs exhibited a maximum absorption at 390 nm (Figure 1), which concurs with a previous report [17,18,19], and particle sizes were ~5 nm [17,18,19,24]. To produce UV radiation, we used a universal handy-type UV lamp frequently employed for the detection of ethidium bromide-stained DNA in agarose gel electrophoresis. The UV that the lamp was able to generate has a wavelength of 365 nm, putting it in the range of UVA (320 to 400 nm), which is safer than other types of UV radiation (i.e., UVC and UVB). UVB (280 to 320 nm) can cause a variety of damaging effects in various biological cells, whereas UVC (200 to 280 nm) is very hazardous to most organisms [25,26].

**Figure 2:**
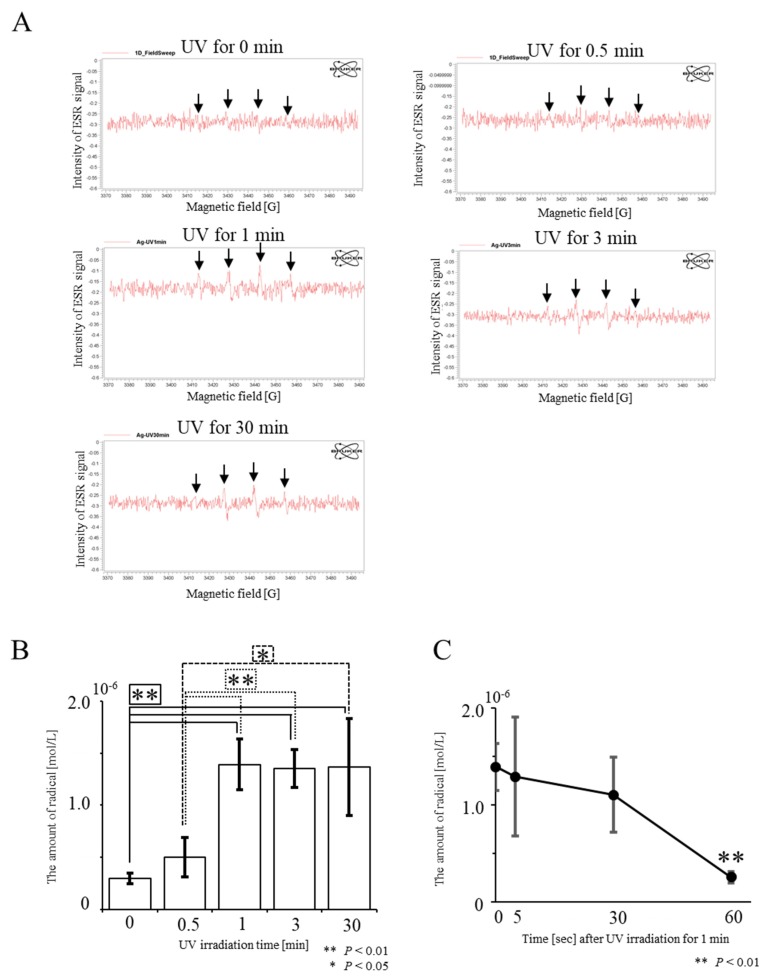
Detection of hydroxyl radicals from UV-irradiated AgNPs using electron paramagnetic resonance (EPR) spectroscopy for the detection of radicals. (**A**) Raw data obtained from AgNPs UV-irradiated for 0, 0.5, 1, 3, or 30 min. In “UV for 0 min”, a very weak peak for hydroxyl radicals is apparent. Four peaks for hydroxyl radicals, indicated by arrows, are discernible in the UV-irradiation groups. (**B**) Evaluation of the amount of hydroxy radicals produced after UV irradiation of AgNPs. The amount of hydroxyl radicals was determined using software solution (Xenon) provided by the Bruker Corporation, from the raw data shown in A. There is no significant difference between “UV for 1 min” and “UV for 3 min”. Experiments were repeated three times on different days. (**C**) Time-dependent decrease in the amounts of hydroxyl radicals generated after “UV for 1 min” at 30 s and thereafter the amount of hydroxyl radicals decreases rapidly.

We first examined whether hydroxyl radicals are generated when a solution containing AgNPs is subjected to UV irradiation. Hydroxyl radicals were detected when a UV-irradiated solution was subjected to an electron paramagnetic resonance (EPR) analysis (Figure 2A). We measured the amounts of hydroxyl radicals in solutions of AgNPs by varying the time of UV irradiation from 0 to 30 min. The presence of hydroxyl radicals was discernible after adding AgNPs to Dulbecco’s modified phosphate-buffered saline without Ca^2+^ and Mg^2+^, pH 7.2 (PBS) (“0 min required for UV irradiation” in Figure 2B). Similar observations were made by Zhang et al. [27], who detected the presence of oxidative stress in natural samples, observing small amounts of hydroxyl radicals in aqueous AgNPs solutions. We observed UV irradiation-mediated enhanced production of hydroxyl radicals from AgNPs. The proportion of hydroxyl radicals increased as UV irradiation continued for up to 1 min and subsequently plateaued (Figure 2B). The amounts of hydroxyl radicals generated by UV irradiation for 1 and 3 min did not differ significantly. The lifespan of hydroxyl radicals generated was very short, at around 30 s (Figure 2C).

There are several possible mechanisms underlying the generation of hydroxyl radicals induced by UV irradiation of AgNPs. An SPR-mediated collective oscillation of conduction electrons may be generated on the surface of UV-irradiated AgNPs, leading to local generation of an enhanced electric field. Homolytic cleavage, a process in which the covalent bonds of water surrounding AgNPs are broken, may occur in response to the collective oscillation of conduction electrons, resulting in the generation of unpaired electrons as well as radicals. Unfortunately, the current results do not support this hypothesis, and further analysis is required to elucidate the mechanism of action.

As described in the Introduction, some reports claim that AgNPs yield ROS, which act as oxidative stressors to kill various types of bacteria [28,29,30,31]. However, these reports appear to rely on the indirect action of Ag, in which antibacterial activity might be elicited through oxidized peripheral substances or modified materials. Electron paramagnetic resonance spectroscopy is considered one of the standard methods for the quantification of ROS; however, the equipment is very expensive [32]. To our knowledge, there has only been one report indicating that this equipment is useful to detect radicals [27]. In this study, we demonstrated the usefulness of an EPR spectroscopy-based direct detection approach. Our present study differs from the experiments of Zhang et al. [27], who explored the mechanism by which ROS exerts microbicidal activity. We observed a 1.7-fold enhanced production of hydroxyl radicals when AgNPs were irradiated for 0.5 min, and 4.5-fold enhanced production when AgNPs were UV-irradiated for 1 min (Figure 2B). The increased levels of hydroxyl radicals should reflect enhanced microbicidal activity of AgNPs.

### 2.2. Antibacterial Activity of UV-Irradiated AgNPs

**Figure 3:**
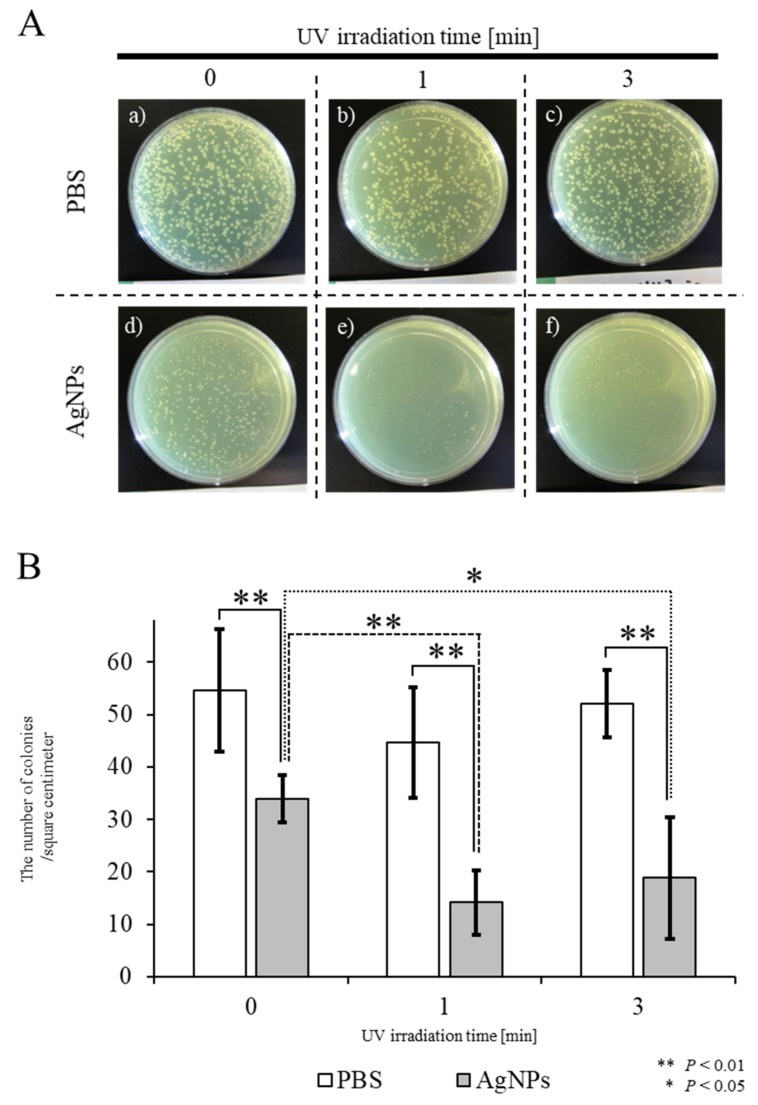
Bactericidal effects of UV-irradiated AgNPs on the survival of *E. coli*. (**A**) Appearance of *E. coli* colonies on agar plates after overnight incubation at 37 °C. Aqueous solutions of PBS or solutions containing AgNPs were subjected to UV irradiation for 0, 1, or 3 min, then each solution was mixed with *E. coli* prior to plating on agar plates. (**B**) Quantitation of colony numbers in each group. In the control PBS groups, shown as white columns, there was no difference in the survival of *E. coli* with or without UV irradiation. In the experimental groups, shown as gray columns, there was a slight decrease in the number of colonies after incubation with non-UV-irradiated AgNPs (designated as “UV for 0 min”). A significant decrease in the number of colonies was observed when *E. coli* were incubated with AgNPs UV-irradiated for one or three min, designated as “UV for 1 min” and “UV for 3 min”, respectively. Experiments were repeated three times on different days.

**Figure 4:**
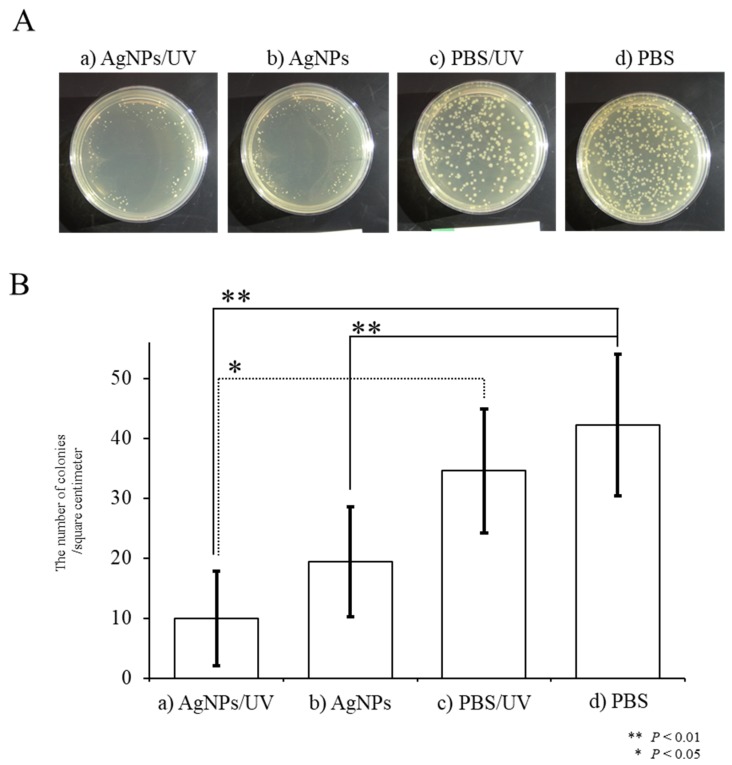
Bactericidal effects of hydroxyl radicals generated in situ on the survival of *E. coli*. (**A**) Appearance of *E. coli* colonies on agar plates after overnight incubation at 37 °C. A solution containing AgNPs was plated onto the surface of agar plates. Then, *E. coli* were seeded onto the surface of agar plates pre-coated with AgNPs, and the surface was subjected to UV exposure for one min (designated as “AgNPs/UV”) or were not irradiated (designated as “AgNPs”). Alternatively, *E. coli* were plated onto non-coated agar plates, and then the surfaces were either subjected to UV exposure for one min (designated as “PBS/UV”) or were not irradiated (designated as “PBS”). (**B**) Quantitation of colony numbers in each group. The presence of AgNPs reduced the number of *E. coli* colonies. The growth of *E. coli* was significantly suppressed when a plate was UV-irradiated for one min. Experiments were repeated three times on different days.

We investigated whether UV-irradiated AgNPs inhibit the survival of the bacterium *E. coli*. Experiments were carried out using two approaches: In the first set of experiments, solutions containing AgNPs were UV-irradiated, and then mixed with *E. coli* prior to seeding onto agar plates (Figure 3). In the second approach, agar plates that had their surface coated with AgNPs were irradiated with UV and plated with *E. coil* (Figure 4).

In the first approach, PBS or PBS-containing AgNPs (PBS/AgNPs) was subjected to UV irradiation for 0, 1, or 3 min, after which, each solution was mixed with *E. coli* prior to plating onto agar plates. After overnight incubation at 37 °C, the number of *E. coli* colonies growing on plates coated with the AgNPs, which had been UV-irradiated for 1 min (Figure 3A-e,B), was significantly lower than that of untreated *E. coli* colonies (“UV irradiation for 1 min” in Figure 3A-b,B). Similar results were observed when UV irradiation was performed for 3 min (“UV irradiation for 3 min” in Figure 3A-f,B). The number of *E. coli* colonies grown in the “AgNPs” group that had not been irradiated was lower than that of those grown in the “PBS” group (PBS vs. AgNPs in Figure 3A-a,d,B). When *E. coli* were grown on plates coated with PBS, the number of *E. coli* colonies was not significantly different, even after UV irradiation for 3 min (Figure 3A-a–c,B). These results indicate that the antibacterial effects of AgNPs were significantly enhanced after exposure to UV for 1 min. This experimental system was modeled on sterilization by spraying chemicals. In this context, radicals generated from AgNPs after UV irradiation could be an alternative to the use of chemicals.

In the second approach, in an experimental group designated as “AgNPs/UV”, *E. coli* were first plated onto agar plate surfaces pre-coated with AgNPs, after which the surfaces were subjected to UV exposure for 1 min (“AgNPs/UV” group). As controls, *E. coli* were first plated onto agar plate surfaces pre-coated with PBS, after which the surfaces were subjected to UV exposure for 1 min (the “PBS/UV” group) or were not UV-irradiated (the “PBS” group). As an additional control, *E. coli* were plated onto agar plates pre-coated with AgNPs, which were not subsequently UV-irradiated (“AgNPs” group). In the AgNPs/UV group, the growth of *E. coli* was suppressed (Figure 4A-a) compared with that of *E. coli* colonies in the other groups (a vs. c and d in Figure 4A,B). There were statistical differences between the PBS and AgNPs/UV groups (*P* = 0.0009) and between the PBS/UV and AgNPs/UV groups (*P* = 0.002) (Figure 4B). Additionally, as in the first experiment, the number of *E. coli* colonies in the AgNPs was lower than that of “PBS” group (*P* = 0.009; b vs. d in Figure 4A,B). These results suggest that the generation of radicals from AgNPs is accelerated after UV irradiation, leading to increased toxicity against *E. coli*. In this study, AgNPs were transiently kept under dry conditions, which was different from the situation in the first approach. As bacteria live in an environment enriched with water, the radicals provided by AgNPs might affect their survival. Alternatively, the survival of bacteria may be influenced by direct contact with AgNPs in the absence of water. In any event, complex materials coated with AgNPs can bring effective bactericidal effects.

Our results indicate that UV irradiation of materials coated with AgNPs produces increased antibacterial activity. The UV radiation used in this study was produced by a universal handy-type UV lamp capable of generating UV of 365 nm, which is within the range of UVA (320 to 400 nm) and is safer than other types of rays such as UVC (200 to 280 nm). UVC is highly biotoxic, in addition to its strong bactericidal action. In contrast, UVA is not only known to be involved in reducing skin elasticity and promoting aging, but also facilitates the exchange of intracellular substances, leading to promotion of cell’s metabolism [26]. Ninety-nine percent of UV rays reaching the earth’s surface from the sun are UVA. This is advantageous, especially when people wearing AgNP-coated protective clothing and medical masks continue to work outside without removing the clothes or masks, because their sterility can be maintained. This UV-enhanced AgNP-based microbicidal system is also useful for protecting against infection in case of occasional unintended contact when healthcare workers change clothes.

## 3. Materials and Methods

### 3.1. Preparation of AgNPs

A suspension of AgNPs (approximately 5 nm, 10 ng/µL, and pH7.2) was prepared as previously described [20,24]. One gram of silver-containing glass powder was dispersed in 100 mL of an aqueous solution of 0.8 wt% glucose in a 500 mL glass vial. The mixture was autoclaved at 121 °C at 200 kPa for 20 min, and then gradually cool to room temperature (~25 °C). The mixture was then centrifuged at 1500 rpm for 10 min at 25 °C, and the AgNPs were collected from the supernatant. To confirm the existence of the AgNPs, UV–Vis spectrometer analysis was performed. The AgNPs were stored in the dark at 4 °C.

### 3.2. Measurement of Radicals from UV-Irradiated AgNPs

Fifty microliters (10 ng/µL) of the AgNPs was subjected to UV (365 nm) irradiation for 0.5, 1, 3, or 30 min. UV irradiation was performed using a Handheld UV Lamp (UVGL-58; Analytik Jena, CA, USA), which is generally used for the detection of DNA. After irradiation with UV, 10 µL of radical trapping reagent 5,5-dimethyl-1-pyrroline N-oxide (DMPO) (#LM-2110; Dojindo Laboratories, Kumamoto, Japan) was added to each sample followed by gentle mixing. Subsequently, the mixture was transferred to a 50 µL calibrated pipet (#2-000-050; Drummond Scientific Company, Pennsylvania, USA) and analyzed using a benchtop EPR (EMX-nano, Bruker Corporation, Massachusetts, USA) together with its proprietary software solution (Xenon). In this study, all experiments were performed in the same area where there was no external light.

### 3.3. Bactericidal Activity of UV-Irradiated AgNPs

The bactericidal activity of the UV-irradiated AgNPs was evaluated as previously reported [18], with a few modifications. *E. coli* (DH5; Takara Bio Inc., Shiga, Japan) were pre-cultured in 2 mL of LB medium and grown at 37 °C for 6 h until reaching an optical density at 600 nm (OD600) of 0.260. The *E. coli* cultures were diluted ten-fold with LB broth, and 100 µL of the diluted *E. coli* suspension was used in each study.

To examine the system that generates radicals in an aqueous solution from AgNPs and then sterilizes *E. coli* with the radicals, 30 µL of AgNP solution was irradiated using UV at a wavelength of 365 nm for either 1 or 3 min, then added immediately to the diluted *E. coli* suspensions. After 1 min, the mixture was plated onto agar plates and subsequently cultured overnight in a 37 °C incubator.

To investigate the bactericidal effect of the AgNP-coated plates on *E. coli*, 100 µL of AgNPs was plated on agar and left to air dry for 5 min at room temperature (~25 °C). The diluted *E. coli* suspension was then plated on agar and subjected to UV irradiation with a wavelength of 365 nm for 1 min. The culture plate was then incubated overnight at 37 °C.

After incubation, the plates were photographed (Cyber-shot; SONY Corp., Tokyo, Japan) and the number of colonies per square centimeter was counted. Bactericidal activity was evaluated from the number of colonies. As a control, PBS solution was used instead of AgNPs. The treatment with AgNPs after sterilization using an autoclave was performed on a clean bench. Aseptic conditions were stringently maintained during the plating of *E. coli* onto agar plates.

### 3.4. Statistical Analysis

The amount of hydroxyl radicals and the number of colonies in each examination are presented as mean ± standard deviation. Statistical analysis was performed using an unpaired *t*-test and one-way factorial analysis of variance. Scheffe’s post hoc test was used for multiple comparisons. The *p*-values were calculated using the JMP14 for Windows software (SAS Institute Inc., North Carolina, USA). A *p*-value of less than 0.05 was considered to indicate statistical significance. Values of *p* less than 0.001 are marked with a double asterisk those less than 0.05 with a single asterisk.

## 4. Conclusions

AgNPs have been shown to have bactericidal action. In this study, we demonstrated that UV irradiation of AgNPs is effective at enhancing their activity. This bactericidal effect is attributable to the UV irradiation-mediated enhanced production of highly reactive hydroxyl radicals generated from AgNPs. The method of UV irradiation is very simple. The UV radiation used in this study was UVA, which is safer for humans than UVC rays and has stronger bactericidal activity. Although challenges persist regarding the effects of AgNPs on human health and the elucidation of the molecular mechanisms underlying the generation of radicals, our findings would contribute to the development of medical materials for further protection against infection. For the practical use of AgNPs, investigating the effects of particle size, formulation, and the stabilizers (added for preventing aggregation of particles) on the behavior of nanometals might be important. Furthermore, if the behavior of nanometals is associated with SPR, the efficiency for radical generation may increase by changing the wavelength of the UV. Biosafety issues and environmental safety concerning AgNPs have also been a subject for discussion [22,33]. These concerns must be carefully addressed before putting AgNPs to practical use. Hydroxyl radicals, renowned as powerful oxidizing agents, are likely to be effective against viruses, bacteria, and in the degradation of chemicals. Therefore, our system may be valuable for the protection of healthcare workers from infection with pathogens such as COVID-19 and SARS, and in the prevention of outbreaks of similar diseases.

## Abbreviations

AgNPs	silver nanoparticles
COVID-19	coronavirus disease 2019
DMPO	5,5-dimethyl-1-pyrroline N-oxide
*E. coli*	*Escherichia coli*
EPR	electron paramagnetic resonance
HIV-1	human immunodeficiency virus-1
HSV	herpes simplex virus
MRSA	methicillin-resistant *Staphylococcus aureus*
NADH	nicotinamide adenine dinucleotide
PBS	Dulbecco’s modified phosphate-buffered saline without Ca^2+^ and Mg^2+^, pH 7.2
ROS	reactive oxygen species
SARS	severe acute respiratory syndrome
SPR	surface plasmon resonance
UV	ultraviolet
UVA	ultraviolet A (320 to 400 nm)
UVB	ultraviolet B (280 to 320 nm)
UVC	ultraviolet C (200 to 280 nm)
